# Deaths from Tick-Borne Encephalitis, Sweden

**DOI:** 10.3201/eid2807.220010

**Published:** 2022-07

**Authors:** Renata Varnaitė, Sara Gredmark-Russ, Jonas Klingström

**Affiliations:** Karolinska Institutet, Stockholm, Sweden (R. Varnaitė, S. Gredmark-Russ, J. Klingström);; Karolinska University Hospital, Stockholm (S. Gredmark-Russ);; Umeå University, Umeå, Sweden (S. Gredmark-Russ)

**Keywords:** tick-borne encephalitis, TBE, ticks, case-fatality rate, standardized mortality ratio, vector-borne infections, viruses, Sweden

## Abstract

We assessed standardized mortality ratio in tick-borne encephalitis (TBE) in Sweden, 2004–2017. Standardized mortality ratio for TBE was 3.96 (95% CI 2.55–5.90); no cases in patients <40 years of age were fatal. These results underscore the need for further vaccination efforts in populations at risk for TBE.

Tick-borne encephalitis (TBE) is caused by the TBE virus (TBEV), which is transmitted to humans via infected ticks or, on rare occasions, via ingestion of contaminated milk from an infected animal (*1*). TBE is endemic in parts of Asia and Europe, including Sweden. TBEV has 3 subtypes: European, Siberian, and Far Eastern (*2*). The European subtype of TBEV is the only known subtype in Sweden (*1*,*3*). TBE is typically a biphasic disease manifesting with febrile influenza-like illness during the first phase, followed by a second phase of neurologic symptoms of different severity, ranging from meningitis to severe meningoencephalitis (*4*). Long-term sequelae are common and a case-fatality rate (CFR) of 0.5% has previously been reported in Europe (*5*–*7*).

TBE became a notifiable disease in Sweden in 2004, and from then on, all cases of TBE are reported to the Public Health Agency of Sweden. Advanced age is a risk factor for severe TBE, and CFRs increase with age (*5*,*7*–*9*). However, CFRs do not account for baseline mortality, a particularly important consideration for the elderly population. To investigate the relative contribution of TBE to overall mortality rates in Sweden, we performed a case–control study and calculated standardized mortality ratio (SMR) for TBE-diagnosed patients during 2004–2017. 

## The Study

Cases of patients with notifiable infectious disease diagnoses in Sweden are reported to the Public Health Agency of Sweden. This case–control study relies on 3 data sources: TBE cases reported to the Public Health Agency of Sweden during 2004–2017; Swedish population register from Statistics Sweden; and the Swedish National Board of Health and Welfare’s Cause of Death Register. The Regional Ethical Review Board in Stockholm, Sweden, approved the study.

**Figure 1:**
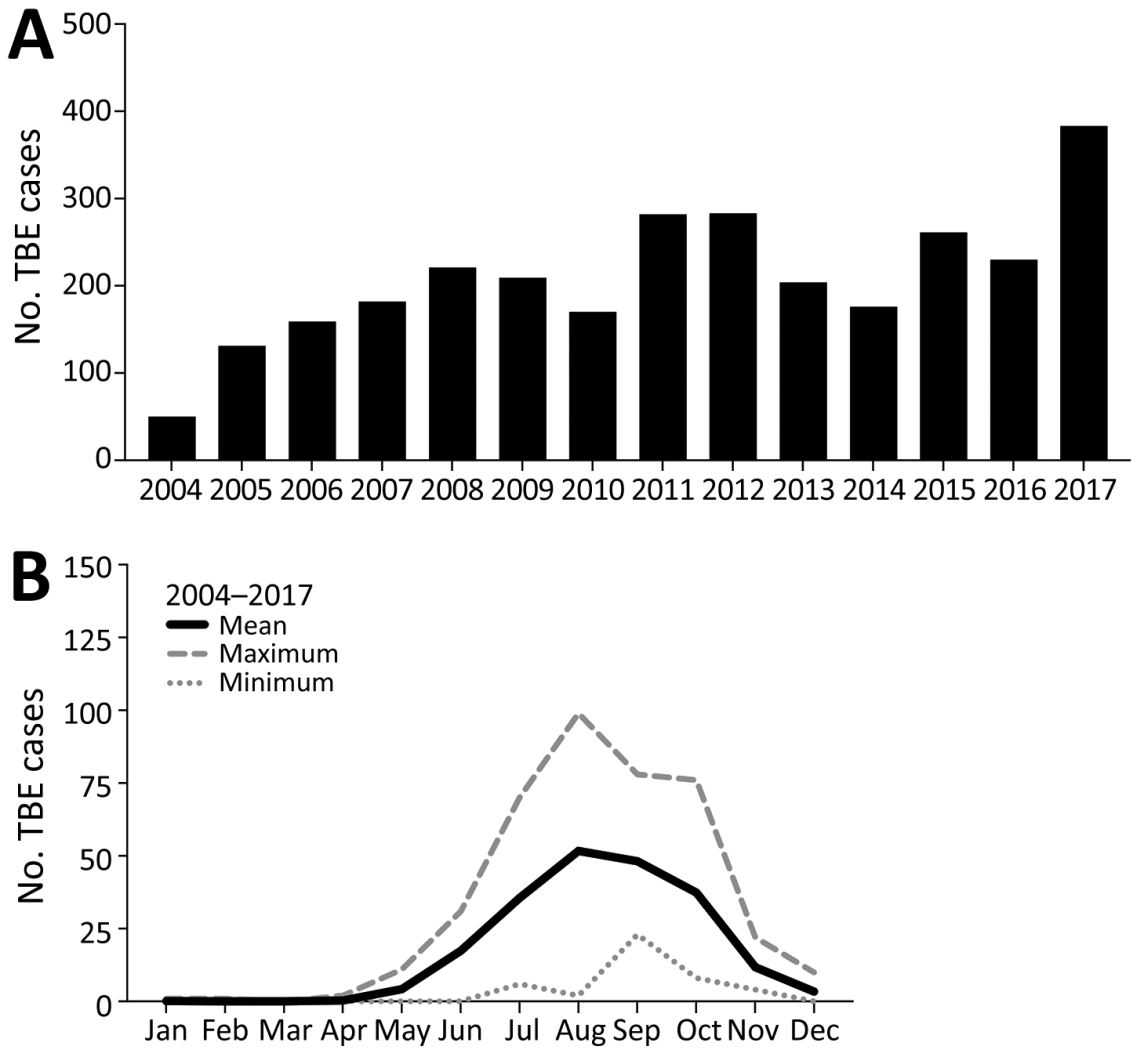
TBE cases reported to the Public Health Agency of Sweden, 2004–2017. A) All cases. B) Mean number of cases reported by month, with minimum (dotted line) and maximum (dashed line) numbers shown. TBE, tick-borne encephalitis.

**Figure 2:**
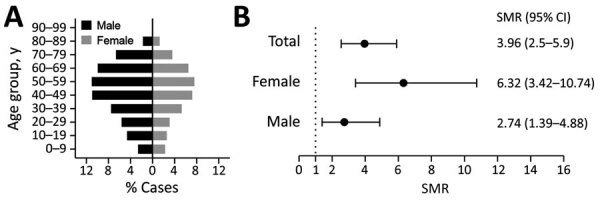
Age and distribution of TBE cases reported to the Public Health Agency of Sweden, 2004–2017. A) Percentages of all cases by sex and age group. B) SMRs of cases by sex. Bars indicate 95% CI within 90 days after the reporting date. SMR, standardized mortality ratio.

We included all 2,941 TBE cases that were reported to the Public Health Agency of Sweden during July 1, 2004–December 31, 2017 (Table). The number of annual TBE cases has gradually increased during 2004–2017; TBE incidence followed a typical seasonal pattern of 90% of all cases reported during June–October (Figure 1, panels A, B). TBE was reported in all age groups; 53% of the cases were in the age group of 40–69 years (Table; Figure 2, panel A). Sixty percent of all reported TBE patients were male (n = 1,777) and 40% female (n = 1,164) (Table; Figure 2, panel A). The median age at diagnosis was 48 (IQR 33–63) years for male patients, 49 (33–61) years for female patients, and 48 (33–62) years for all TBE patients.

**Table:** Characteristics of TBE cases reported to the Public Health Agency of Sweden during 2004–2017*

Group	No. (%)	Case-fatality rate, %	Mortality rate in controls, %	No. observed deaths, total, M/F	Expected deaths	SMR (95% CI)	p value
Total cases	2,941 (100)	0.75	0.19	22	5.55	**3.96 (2.55–5.9)**	**<0.001**
Sex							
M	1,777 (60)	0.56	0.21	10	3.65	**2.74 (1.39–4.88)**	**0.006**
F	1,164 (40)	1.03	0.16	12	1.90	**6.32 (3.42–10.74)**	**<0.001**
Age group							
0–9	143 (5)	0	0	0	0	NA	NA
10–19	212 (7)	0	0	0	0	NA	NA
20–29	256 (9)	0	0	0	0	NA	NA
30–39	379 (13)	0	0.01	0	0.05	NA	NA
40–49	532 (18)	0.19	0.02	1, 0/1	0.10	10.00 (0.5–49.32)	0.100
50–59	546 (19)	0.37	0.16	2, 0/2	0.85	2.35 (0.39–7.77)	0.264
60–69	480 (16)	1.25	0.22	6, 4/2	1.05	**5.71 (2.32–11.89)**	**<0.001**
70–79	301 (10)	3.32	0.66	10, 3/7	2.00	**5.00 (2.54–8.91)**	**<0.001**
80–89	87 (3)	3.45	1.50	3, 3/0	1.30	2.31 (0.59–6.28)	0.186
90–99	5 (0.2)	0	4.00	0	0.20	NA	NA

*Bold text indicates statistically significant values. NA, not applicable; SMR, standardized mortality ratio (SMR) within 90 d after the reporting date of TBE; TBE, tick-borne encephalitis.

To assess deaths from TBE in Sweden, we measured CFRs and SMR. We matched each reported TBE case (n = 2,941) with 20 control persons from the population register of Sweden on the basis of age, sex, and county of residence (n = 58,820). We then linked TBE cases and matched controls to the Swedish National Cause of Death Register. We identified fatal TBE cases, those in which the patient died <90 days after the reporting date, as well as deaths within the matched control population during the same time period (Appendix Figure).

We found that the overall CFR in TBE during 2004–2017 was 0.75%; in male patients, CFR was 0.56%, and in female patients, 1.03% (Table). CFR increased with age, reaching 3.45% in the 80–89-year age group. Although TBE was reported in all age groups, we noted no fatal cases within 90 days of reporting date in patients <40 years of age (Table).

To account for the baseline mortality rate in the Swedish population, we next measured SMR by dividing the number of deaths in TBE-diagnosed persons by the expected number of deaths estimated from the matched controls (Appendix Figure). We calculated 95% CI for SMRs using mid-P exact test at the Open Source Epidemiologic Statistics for Public Health (*10*). The overall SMR for TBE was 3.96 (95% CI 2.55–5.9; p<0.001) (Table; Figure 2, panel B). We observed higher SMR in female patients (SMR 6.32, 95% CI 3.42–10.74; p<0.001) than male patients (SMR 2.74, 95% CI 1.39–4.88; p = 0.006), which suggested a potential sex-related difference in deaths due to TBE (Table; Figure 2, panel B). When we stratified patients by age, we observed statistically significant SMRs of 5.71 (95% CI 2.32–11.89; p<0.001) in TBE patients 60–69 years of age and 5.00 (95% CI 2.54–8.91; p<0.001) in TBE patients 70–79 years of age (Table).

## Conclusions

In this case–control study, we found the mortality rate in TBE patients in Sweden to be ≈4-fold higher than that of the matched control population. CFR of 0.75% for TBE in this study is comparable with previously reported CFR of 0.5% in Europe (*5*). Although TBEV infection has been reported for all age groups, including children, we found no fatal cases within 90 days after the reporting date in persons <40 years of age. However, in TBE patients >60 years of age, we observed a significantly higher SMR, highlighting the need for further vaccination efforts against TBE, particularly within this age group. This finding is consistent with other studies that reported TBE to be more severe in older patients (*5*,*7*,*11*).

TBE incidence is typically higher in male than female patients; we observed the same pattern over a 14-year period in Sweden (*5*), where 60% of all TBE patients were male. However, despite higher incidence in male patients, we found higher CFRs and SMR in female TBE patients. In several infectious diseases, male patients show higher incidence and have more severe outcomes than female patients (*12*). Our results indicate that there may also be a sex-dependent difference in the outcome of TBE, but because we had a relatively low number of fatal TBE cases in our study, this sex-dependent difference in deaths should be investigated in a larger TBE patient cohort.

A strength of this study is the use of national registers that enabled us to estimate baseline mortality rates in the national population matched to TBE cases by sex, age, and county of residence. SMR, as opposed to CFR, accounts for the baseline mortality rate within a given population subgroup, which is a particularly important consideration when estimating deaths in the elderly population. A limitation of this study is that the controls were matched to TBE cases without taking into consideration lifestyle, socioeconomic status, or comorbidities. Because this disease requires an active lifestyle for exposure, it is possible that TBE patients in Sweden are healthier than the control populations, which could result in the underestimation of SMR in our study. On the other hand, the total deaths from TBE may be overestimated considering that TBEV-infected persons can follow a subclinical course of infection and may not be reported in the healthcare system (*13*).

In summary, we saw a substantially increased SMR for TBE patients in Sweden during 2004–2017 compared with the general population. Our findings highlight the need for further vaccination efforts against this disease, particularly for older persons.

Appendix Additional information about a study of deaths from tick-borne encephalitis in Sweden.
