# Supplementary material for: Deaths from Tick-Borne Encephalitis, Sweden
Source: Emerg Infect Dis. 2022 Jul;28(7):1471–4. doi: 10.3201/eid2807.220010 (PMC9239873; doi:10.3201/eid2807.220010)
Supplement: Appendix — Additional information about a study of deaths from tick-borne encephalitis in Sweden. [file 22-0010-Techapp-s1.pdf]

Article DOI: https://doi.org/10.3201/eid2807.220010

Deaths from Tick-Borne Encephalitis, Sweden

Appendix

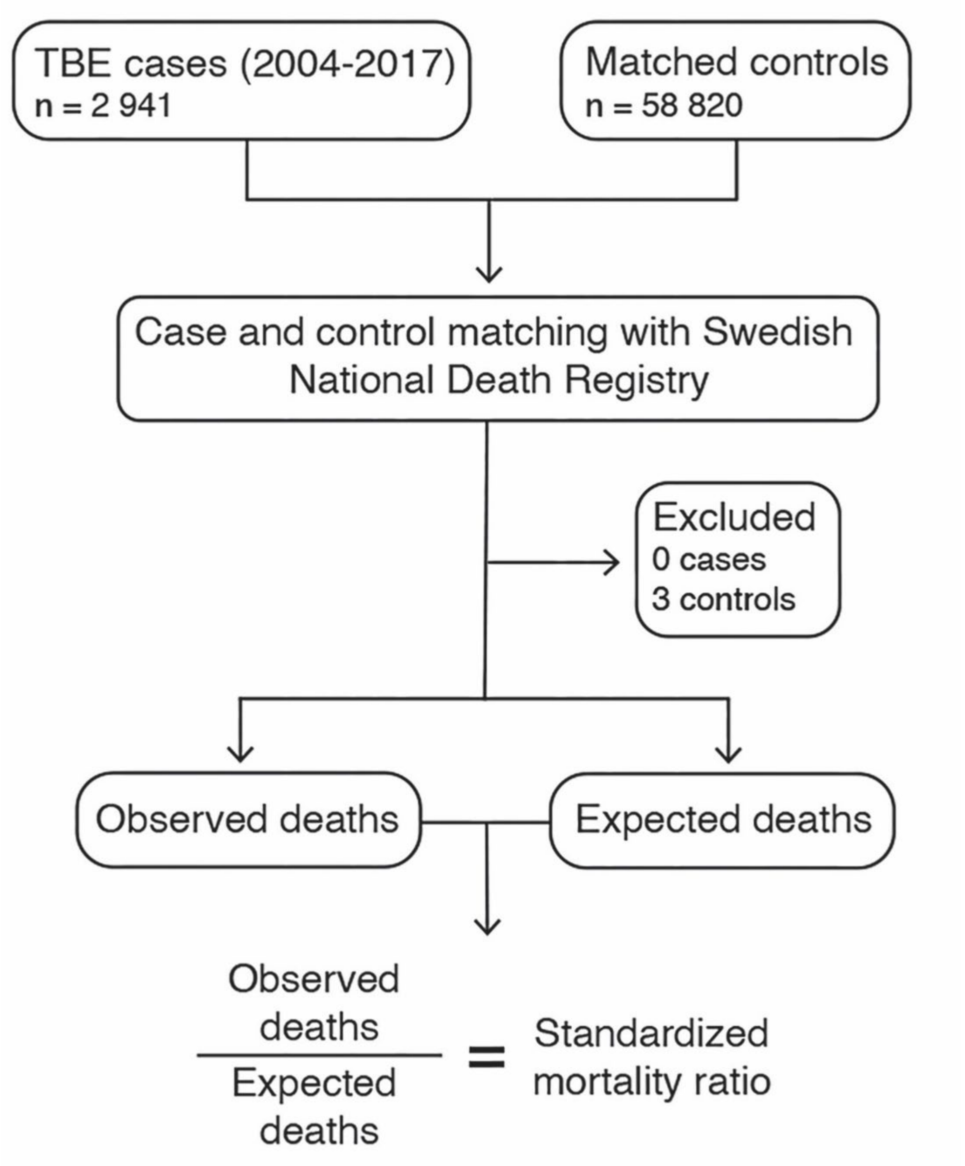

Appendix Figure. Flow chart showing study design for a study of deaths from tick-borne encephalitis in Sweden, 2004–2017.
